# Autonomic nervous system and cardiac neuro-signaling pathway modulation in cardiovascular disorders and Alzheimer’s disease

**DOI:** 10.3389/fphys.2023.1060666

**Published:** 2023-01-30

**Authors:** Andrea Elia, Silvia Fossati

**Affiliations:** Department of Neural Sciences, Alzheimer’s Center at Temple (ACT), Lewis Katz School of Medicine at Temple University, Philadelphia, PA, United States

**Keywords:** neurotrophic factors, autonomic nervous system, cardiac innervation, neurochemical pathways, Alzheimer’s disease, heart failure, myocardial infarction

## Abstract

The heart is a functional syncytium controlled by a delicate and sophisticated balance ensured by the tight coordination of its several cell subpopulations. Accordingly, cardiomyocytes together with the surrounding microenvironment participate in the heart tissue homeostasis. In the right atrium, the sinoatrial nodal cells regulate the cardiac impulse propagation through cardiomyocytes, thus ensuring the maintenance of the electric network in the heart tissue. Notably, the central nervous system (CNS) modulates the cardiac rhythm through the two limbs of the autonomic nervous system (ANS): the parasympathetic and sympathetic compartments. The autonomic nervous system exerts non-voluntary effects on different peripheral organs. The main neuromodulator of the Sympathetic Nervous System (SNS) is norepinephrine, while the principal neurotransmitter of the Parasympathetic Nervous System (PNS) is acetylcholine. Through these two main neurohormones, the ANS can gradually regulate cardiac, vascular, visceral, and glandular functions by turning on one of its two branches (adrenergic and/or cholinergic), which exert opposite effects on targeted organs. Besides these neuromodulators, the cardiac nervous system is ruled by specific neuropeptides (neurotrophic factors) that help to preserve innervation homeostasis through the myocardial layers (from epicardium to endocardium). Interestingly, the dysregulation of this neuro-signaling pathway may expose the cardiac tissue to severe disorders of different etiology and nature. Specifically, a maladaptive remodeling of the cardiac nervous system may culminate in a progressive loss of neurotrophins, thus leading to severe myocardial denervation, as observed in different cardiometabolic and neurodegenerative diseases (myocardial infarction, heart failure, Alzheimer’s disease). This review analyzes the current knowledge on the pathophysiological processes involved in cardiac nervous system impairment from the perspectives of both cardiac disorders and a widely diffused and devastating neurodegenerative disorder, Alzheimer’s disease, proposing a relationship between neurodegeneration, loss of neurotrophic factors, and cardiac nervous system impairment. This overview is conducive to a more comprehensive understanding of the process of cardiac neuro-signaling dysfunction, while bringing to light potential therapeutic scenarios to correct or delay the adverse cardiovascular remodeling, thus improving the cardiac prognosis and quality of life in patients with heart or neurodegenerative disorders.

## 1 Introduction

The sinoatrial node, in the wall of the heart’s right atrium, is the independent cardiac pacemaker; it originates the cardiac impulse, whose diffusion through cardiomyocytes is allowed by their tight junctions, forming a functional syncytium. The central nervous system regulates the cardiac rhythm, modulating its speed through the two branches of the autonomic nervous system: the sympathetic system, which exerts its function through norepinephrine (NE, a key marker of the adrenergic system), and the cholinergic system, whose neuromodulator is acetylcholine (ACh). In addition, the cardiac innervation undergoes a characteristic regulation by neuro-signaling: small neuroactive molecules (neurotrophins) contribute to producing an innervation gradient through the membranes of the myocardium (epicardium to endocardium). The impairment of this neurochemical pathway occurs in different cardiometabolic and neurodegenerative disorders and may cause dysfunction in heart rhythm, leading to severe denervation or sudden cardiac death (SCD), as shown in myocardial infarction, heart failure, and possibly, Alzheimer’s disease (AD). Here, we explore the physiological and molecular mechanisms involved in the alterations of the heart neuronal network observed both in cardiac and neurodegenerative illnesses and we propose therapeutic solutions for cardiac pathologies with a recognized imbalance in the two components of the autonomic system responsible for cardiac remodeling.

## 2 Anatomical and physiological characteristics of the cardiac autonomic system

The control of cardiac activity is (Langley, 1922; Silva et al., 2017) directly exercised through the sympathetic and the parasympathetic nervous systems (Rossi and Nappo, 1994), which play a key role in chronotropic, inotropic, dromotropic, and lusitropic effects; in particular, norepinephrine and epinephrine increase cardiac output, whereas acetylcholine reduces it. In resting conditions, vagal tone prevails on the sympathetic one. Circulating catecholamines, released by neurons and produced by the adrenal medulla (epinephrine 80% and norepinephrine 20%), exert a sympathetic action on the heart, activating the prevalent adrenergic receptor population localized on cardiomyocytes: β1-adrenergic receptors (β1- ARs), which couple to stimulatory G proteins. The resulting effects are an increase in slow calcium entry current (Ca^2+^), which produces inotropic positive effects; enhanced potassium currents (K^+^) (Mesirca et al., 2013), which maintain the cardiac membrane electrical potential; and a reduction in the sinus node pacemaker potential threshold, with positive chronotropic effects.

Norepinephrine also provokes a vasoconstriction effect, triggering the α-1 receptors (Pellegrini et al., 1994) on vascular smooth muscle cells. The enhancement of peripheral vascular resistance and blood pressure, in turn, results in both aortic and carotid sinus baroreceptors activation.

The Autonomic Nervous System exerts multiple non-voluntary effects on different peripheral organs. Neuronal cells of the ANS are localized in the autonomic ganglia. After they synapse with preganglionic neurons, the postganglionic axons activate effector organs. Sympathetic preganglionic neurons (SPNs) involved in cardiovascular regulation are located in the intermediolateral cell column (IML) of the lateral horn and gray commissure along T1–L2 spinal levels. The SPNs between T1–4 are essential for cardiac sympathetic modulation (Gilbey and Michael Spyer, 1993; Waxenbaum et al., 2022). Postganglionic efferent fibers extend from the intrathoracic ganglia to the atrial and ventricular tissue, crucial elements of the cardiac conduction network, spanning mainly as mixed cardiopulmonary nerves (Armour and Hopkins, 1981; Hopkins and Armour, 1984; Kawashima, 2005). In general, SPNs act via acetylcholine release, activating nicotinic acetylcholine receptors, while postganglionic neurons secrete norepinephrine. Albeit norepinephrine represents the leading adrenergic neurotransmitter, neuropeptide Y (NY) and galanin are also implicated in the neuronal activity released from postganglionic nerve endings (Lundberg et al., 1990; Hoover et al., 2009; Herring et al., 2012). These neuromolecules may regulate additional peripheral sympathetic functions, which are dysregulated in cardiac diseases, such as heart failure (HF) (Shanks and Herring, 2013; Ajijola et al., 2020). The SNS regulates cardiac activity through chronotropic, inotropic, dromotropic, and lusitropic effects (Burwash et al., 1993; Fukuda et al., 2015). Of note, a local control of cardiac electrical activity was observed in the porcine model, due to reduced differential of activation recovery intervals in different areas of the left ventricle (LV) (Yanowitz et al., 1966; Vaseghi et al., 2012; Vaseghi et al., 2013). Yet, in a myocardial post-ischemic *in vivo* model, this local control of cardiac electrical activity is altered, accompanied by a higher difference in myocardial repolarization and impaired activation propagation (Ajijola et al., 2013).

### 2.1 Adrenergic system remodeling in cardiac disorders

Along this line, the adrenergic nervous system’s adverse remodeling has been well characterized in different cardiovascular disorders [i.e., myocardial infarction (MI), HF] (Cao et al., 2000a; Zhou et al., 2004; Ajijola et al., 2012; Ajijola et al., 2015). Moreover, myocardial dysfunction gradually leads to an electrical excitability impairment, with neuromodulator dysregulation and cardiac nerve fibers impoverishment (Cao et al., 2000a; Gardner et al., 2015; Tapa et al., 2020). Furthermore, the ischemic process in the cardiac tissue increases oxidative stress, along with adenosine and other cardiolesive mediators (such as inflammatory cytokines), resulting in the activation of myocardial afferent neurons (Malliani et al., 1973; Dibner-Dunlap et al., 1993; Minisi and Thames, 1993; Huang et al., 1996). Interestingly, the sympathoexcitation reflex may partially attenuate the detrimental ischemic remodeling of the working myocardium (Minisi and Thames, 1991; Schwartz et al., 1992; Ardell et al., 2019). Histologic modifications in stellate ganglia neurons were found both in humans with ischemic cardiomyopathy, as well as in porcine models of chronic ischemia, resulting in inflammation enhancement, glial cell stimulation, and an increase in reactive oxygen species (ROS) production (Ajijola et al., 2012; Ajijola et al., 2015). Clinically, sympathetic hyperactivation in heart failure is treated pharmacologically through beta-adrenergic receptor (β-AR) antagonists and renin-angiotensin-aldosterone system (RAAS) inhibitors (Airaksinen et al., 1999; Floras, 2009; Triposkiadis et al., 2009; Fukuda et al., 2015). Additionally, neuromodulatory strategies to reduce sympathetic hyper-tone, (e.g., cardiac adrenergic denervation, thoracic epidural anesthesia, renal denervation, and stellate ganglion blockade) have been used in heart disease patients (Triposkiadis et al., 2009; Bourke et al., 2010; Fukuda et al., 2015; Shivkumar et al., 2016). Research studies also suggest the application of kilohertz frequency alternating current to the adrenergic chain, which attenuates sympathetic firing to the cardiac tissue, and reversibly blocks adrenergic conduction (Buckley et al., 2017). Similarly, in a post-MI experimental model, Chui et al. demonstrated that reversibly blocking adrenergic stimulation is associated with a decreased risk of ventricular tachycardia/ventricular fibrillation (Chui et al., 2017). Given the growing interest in neuromodulatory applications as a therapeutic solution for cardiovascular diseases, and the increased risk to develop neurodegenerative pathologies, such as AD, in patients with cardiovascular diseases, these innovative strategies and the associated molecular mechanisms deserve a deeper investigation.

### 2.2 Parasympathetic control of cardiovascular activity

The intrinsic cardiac nervous system (ICNS) is characterized by clusters of ganglia known as ganglionated plexi, localized in epicardial fat pads (Armour, 2008; Ardell et al., 2016). These ganglionated plexi are composed of a wide population of neurons, including cholinergic neurons and those that receive parasympathetic afferents, neurons that receive afferent signals directly from cardiomyocytes, and local interneurons (Armour, 2008; Ardell et al., 2016). Moreover, the ICNS interacts with upstream neural stations to control the electromechanical activity of the heart, beat by beat (Cardinal et al., 2009). However, maladaptive nervous system remodeling, found in myocardial infarction, heart failure, and other cardiac conditions, may affect the homeostasis of the ICNS dynamic neural system (Hardwick et al., 2008; Beaumont et al., 2015; Rajendran et al., 2016). Notably, Rajendran and others, described the processing dysfunction of afferent and efferent ICNS neuronal inputs in a cardiac post-ischemic porcine model (Rajendran et al., 2016). Interestingly, an analogous impairment also affects the intrathoracic and primary sensory ganglia in humans and animal models (Zucker et al., 2012; Wang et al., 2014; Ardell et al., 2016; Ajijola et al., 2017; Yoshie et al., 2020).

The cardiac plexus is situated at the base of the heart and is composed of a combination of cardiac nerves and small parasympathetic ganglia. Among these ganglia, the most important is the Wrisberg ganglion, located between the tracheal bifurcation and the pulmonary artery division. In addition, other small intrinsic parasympathetic ganglia are in the cardiac atrial wall. From the cardiac plexus, some nerve fibers run along the right coronary artery, left coronary artery, and their branches, reaching the heart. Other fibers penetrate the sinoatrial and atrioventricular node, or project to the thicker vascular walls in the atrial and ventricular myocardium. Stimulation of the vagus motor nucleus reduces both heart rate and myocardial contraction with a similar effect upon pressure. The vagus nerve is the 10th and longest cranial nerve, which provides an indispensable brain-body connection that modulates essential aspects of autonomic physiology like heart rate, breathing, blood pressure, gut motility, reflexes, and vital behavior (Prescott and Liberles, 2022). Vagal afferents provide the predominant sensory innervation to the heart, aorta, and other large caliber vessels, through the aortic depressor nerve. This nerve emerges from the vagal trunk and reaches the aortic arch and the right subclavian artery, close to its bifurcation with the common carotid artery, where the two leading vagal sensory neurons are located (Prescott and Liberles, 2022). These two nerve centers are involved in the neuronal innervation of the vasculature, monitoring blood flow along the whole circulatory system. Interestingly, sensory neurons are greatly affected by the freshly oxygenated blood pumped from the left ventricle, thus stabilizing blood pressure gradients and oxygenation rates along the vasculature. Vagal sensory neurons are categorized based on cellular features, such as biogeography, conduction velocity, developmental origin, and/or electrophysiological activity. Besides molecular functions, the physiological characteristics of vagal neurons are influenced by their terminal structures and strategic location along the body. Specifically, different chemosensory neurons react to the same neuromodulators, but are placed close to various upstream sensory cells, thus transmitting different sensory inputs to the brain tissue. Vagal mechanosensory neurons represent first-order sensory neural stations that directly detect internal organ cues (Reimann et al., 2008; Krasteva et al., 2011; Moreno-Domínguez et al., 2020). Conversely, different vagal afferents termed second-order neurons, sense signals from upstream sentinel cells, including enteroendocrine cells, glomus cells, immune cells, and taste cells (Bessac and Jordt, 2008; Han et al., 2018). In sum, the vagus nerve significantly impacts daily physiological functions. Therefore, approaches to modulate its activity may represent an intriguing and innovative opportunity for therapeutic solutions.

Notably, vagotomy (subdiaphragmatic) and vagus nerve electrical stimulation (VNS) seem to represent a potential therapeutic strategy in different clinical conditions, albeit some clinical concerns still remain to be addressed. Understanding vagal sensory activity may open new therapeutic horizons, turning on or off specific afferent endings, thus modulating the function of the autonomic nervous system. Of note, cardiac neurons of the PNS are located in the dorsal motor nucleus of the vagus (DMV) nerve, and the nucleus ambiguous in the medulla oblongata. Cholinergic preganglionic neurons project through the vagus and glossopharyngeal nerves, synapsing with postganglionic cells that in turn synapse onto the heart. Therefore, unlike sympathetic neurons, cardiac parasympathetic fibers are anatomically independent of the spinal cord (Airaksinen et al., 1999; Panneton et al., 2014; Petko and Tadi, 2022; Tindle and Tadi, 2022). The cholinergic system (Herring and Paterson, 2009) performs its function through the vagus nerve and its intrathoracic projections, releasing acetylcholine, nitric oxide, and vasoactive intestinal peptide as neurotransmitters by postganglionic fibers (Prescott and Liberles, 2022). Specifically, acetylcholine operates on inhibitory G protein-coupled muscarinic (Fu et al., 1993) M2 receptors, located on atrioventricular and sinoatrial cells and atrial cardiomyocytes, exerting distinct responses: a chronotropic negative effect (decrease of heart rate) due to the activation of potassium channels that produces sinus node cells hyperpolarization and a dromotropic negative effect (reduction of the impulses coming from the atrioventricular node) (Dhein et al., 2001).

### 2.3 Distribution patterns of sympathetic and parasympathetic nerves

New scientific insights (Chow et al., 2001) demonstrated a parasympathetic distribution both in ventricles and atria compartments with a greater fiber density in the endocardium compared to the epicardium (Crick et al., 1994; Crick et al., 1999). The right ventricle shows higher nerve density than the left one, although the subendocardium of the left ventricle denotes more fiber density than the right ventricle endocardium layer. As emerged by scintigraphy studies with metaiodobenzyl guanidine iodine 123 (MIBG-I (Haïssaguerre et al., 1998), different distribution patterns of sympathetic and parasympathetic nerves may modify cardiac performance under both physiologic and pathologic conditions (Rengo et al., 2016). Myocardial scintigraphy is a diagnostic analysis involved in the identification of myocardial perfusion dysfunction in cardiac stress conditions, often performed to detect ischemic heart disease. MIBG is a norepinephrine analog and one of the most used tracers for single-photon emission tomography (SPECT) and positron emission tomography (PET). MIBG allows for the visualization of presynaptic sympathetic nerve function due to its high affinity for presynaptic norepinephrine uptake-1 (NET) (Asghar et al., 2017), and is therefore a marker for cardiac adrenergic innervation. Studies have also analyzed cardiac fibers’ density with immunohistochemical approaches both in murine models and in human autopsy samples (Vracko et al., 1991; Wink et al., 2020). Kawano et al. investigated the distribution of autonomic nerves in autopsied hearts of humans without cardiovascular disease, using immunolabelling techniques for a cholinergic modulator (acetylcholinesterase, AChE) and an adrenergic marker (tyrosine hydroxylase, TH) (Kawano et al., 2003; Kawashima, 2005). The authors showed a greater prevalence of AChE-positive fibers and TH-positive axons density in the atrial chamber than in the ventricle, and lower innervation at the apex, compared to the ventricular base. Furthermore, more cholinergic positive fibers have been found in the subendocardial thickness than in the myocardium subepicardial layer. Overall, it is evident that the different distribution of adrenergic and cholinergic fibers modulates cardiac activity both in physiological and in pathological conditions.

Conversely, extracardiac signals of the sympathetic and parasympathetic nervous system interplay with a complex network of intrinsic cardiac neurons involved in the epicardial neural plexus formation (Armour, 2004). Notably, through its organization into ganglionated subplexuses on the surface of atria and ventricles (close to both the sinoatrial and atrioventricular nodes) (Pauza et al., 2000), the epicardial neural plexus constitutes a further layer of cardiac modulation of autonomic function (Wickramasinghe and Patel, 2013).

### 2.4 Additional modulators of ANS homeostasis

Of note, besides the autonomic reflexes, ANS homeostasis is modulated by a sophisticated and well-organized cardiovascular neurohormonal axis. Notably, stress-sensitive baroreceptors (mechanoreceptors) are located in both high-pressure arterial (aortic arch and carotid sinus) and low-pressure venous networks (systemic veins and atria/pulmonary arterial interface). Central and peripheral chemoreceptors, present in the brainstem, carotid sinus, and aortic arch respectively, detect the modifications in arterial oxygen and carbon dioxide concentration. Lastly, polymodal receptors located in the walls of the cardiac chambers, respond to both mechanical and chemical inputs and stimulate sympathetic nervous system outputs in the setting of receptor activity desensitization (Triposkiadis et al., 2009). In addition, ANS function is regulated via bidirectional feedback from the renin-angiotensin-aldosterone system. Notably, renal hypoperfusion, liable for cardiac output decreases, induces renin secretion and angiotensin- II (AT- II) production, leading to the enhancement of SNS tone centrally (Goldsmith, 2004; Li et al., 2006a) and abolishing, in parallel, baroreflex-modulated suppression of SNS activity (Murakami et al., 1997; Paton et al., 2013). Conversely, SNS output enhancement induces renin release (Goldsmith, 2004).

## 3 Molecular aspects of cardiac innervation: Neurochemical pathways

Cardiac network fibers are under the control of neuropeptides involved in the differentiation, development, and maturation of neural cell bodies (Kimura et al., 2012) (Table 1). These neurotrophic factors (NTFs) are a family of biomolecules, mostly peptides or small proteins, released from nervous tissue with paracrine and autocrine effects (Patel et al., 2000; Nguyen et al., 2010; Descamps et al., 2018). NTFs (Levi-Montalcini, 1987) support the growth, survival, and differentiation of both developing and mature neurons. Most NTFs (Friedman, 2000) exert their trophic effects on neurons through tropomyosin-kinase (Trk) (Huang and Reichardt, 2003), usually activating A-isoform tyrosine kinase (TrkA) receptors. In the mature nervous system, they stimulate neuronal survival (Francis et al., 1999), induce synaptic plasticity (Hasan, 2013), and modulate the formation of long-term memories. NTFs also encourage the initial growth and development of neurons in the central and peripheral nervous systems, and they can also regenerate damaged neurons as described in *vitro* tests and animal models (Frade and Barde, 1998). The target tissue also liberates some of these neuromodulators to guide the growth of developing axons.

**TABLE 1:** Neurochemical control of cardiovascular disorders.

Disease model	Species	Pathways	Effects	Therapies	References
AF	Human, Rodent, Canine	β–adrenergic stimulation, derangement of the ET-1/NGF pathway	Cardiac autonomic nervous system imbalance, Adrenergic and cholinergic fibers hyper-sprouting, Atria morphological remodeling	β-blockers, Cardiac vagal limb ablation	Swissa et al. (2005), Tan et al. (2008), Xie et al. (2011), Li et al. (2013), Suita et al. (2015)
MI	Human, Rodent, Canine	β–adrenergic hyperstimulation, sinusoidal pattern in NGF expression, inflammatory activity	Early hyperinnervation followed by NGF depletion and myocardial denervation, trans-differentiation of the autonomic cardiac phenotype	β-blockers, ACE inhibitors, NGF receptor (TrkA) antagonist ligands	Kuruvilla et al. (2004), Zhou et al. (2004), Kimura et al. (2010), Shi and Habecker (2012), Li and Li (2015), Olivas et al. (2016)
HF	Human, Rodent, Canine	NE spillover, β–adrenergic signaling dysregulation	Neurotrophic factors decline, TH synthesis inhibition, disruption of cardiac sympathetic fibers, myocardial denervation	β-blockers, ACE inhibitors, Sartans	D'Elia et al. (2014), Lorentz et al. (2013), Krum et al. (2011a), Fowler (2004)
Aging/AD	Human, Rodent	Cerebral Aβ aggregates and tau neurofibrillary tangles, neuroinflammation, cerebral dysfunction, brain neuro-signaling pathway dysregulation	Calcium handling impairment, cardiac chambers dilation, HF	β-blockers, ACE inhibitors, Sartans	Li et al. (2006b), Gianni et al. (2010), Cermakova et al. (2015), Troncone et al. (2016), Sanna et al. (2019), Tublin et al. (2019)

Abbreviations: AF, Atrial Fibrillation; MI, Myocardial Infarction; HF, Heart Failure; AD, Alzheimer’s disease.

Different NTFs have been identified, such as nerve growth factor (NGF), brain-derived neurotrophic factor (BDNF) (Fulgenzi et al., 2015; Mitre et al., 2017), neurotrophin- 3 (NT-3) (Fariñas et al., 1994; Kawaguchi-Manabe et al., 2007), neurotrophin-4/5 (NT-4/5) and glial cell line-derived neurotrophic factor (GDNF) (Airaksinen et al., 1999). One of the main members of the neurotrophins class is NGF, a signal protein encoded by the NGF gene (Levi-Montalcini, 1987), produced during regenerative processes, and implicated in the outgrowth and survival of nerve fibers. This peptide works in concert with BDNF (Lorgis et al., 2009; Mitre et al., 2022), encoded by the BDNF gene, which supports neuronal survival via synaptogenesis and neuronal differentiation. BDNF exerts its functions by binding the B-isoform of tropomyosin-kinase receptor (TrkB) (Feng et al., 2015), involved, among the others, in the regulation of mood, pain sensitivity, memory, and neuronal homeostasis. Neurotrophin-3, encoded by the gene NT-3 (Brennan et al., 1999), is another NTF that has activity on certain neurons of the peripheral and central nervous systems; it ensures the survival and differentiation of existing neurons and facilitates the growth and differentiation of new neurons and synapses, including the cardiac nerve fibers. GDNF is a small human protein encoded by the GDNF gene, which powerfully promotes the survival of many neuronal types. The most prominent feature of GDNF is its ability to sustain the survival of dopaminergic neurons and motor neurons. GDNF is the first member of the GDNF ligands family (GFL), which also includes neurturin (NRTN), artemin (ARTN (Baloh et al., 1998), and persephin (PSPN).

GFLs play a key role in many biological processes including cell survival, neuritogenesis, cell differentiation, and cell migration. These neuropeptides perform their activities through GDNF family receptor-α (GFRα) receptors, particularly GFRα1, and a receptor tyrosine kinase (RET receptor) encoded by the RET proto-oncogene. The signaling complex involves a member of the GFRα protein family (GFR1α) and RET. The activation of the first one induces the initial GFL-GFRα receptor complex formation, then the complex enrolls two molecules of RET, triggering trans-autophosphorylation of specific tyrosine residues within the tyrosine kinase domain of each RET molecule; phosphorylation of these tyrosines promotes intracellular signal transduction processes.

### 3.1 Neurotrophic factors and the cardiac autonomic system

The cardiac autonomic system (Coote and Chauhan, 2016) also undergoes a molecular control regulated by particular neuromodulators, such as NGF and semaphorin 3a (Sema3a) (Ieda et al., 2007), a modulator of the “chemical repulsive factor” group (Tang et al., 2004). These two molecules operate together to support a regular innervation pattern from the epicardial to the endocardial compartment (Jiang et al., 2007). Indeed, an alteration of this model is related to dysfunction of cardiac signaling and may cause fatal arrhythmias (Chen et al., 2007).

Different reports (Kimura et al., 2012) have shown that sympathetic fibers release NGF, which promotes their survival and differentiation; in physiological conditions, this marker has been identified in different compartments, especially in those deeply innervated. It has also been detected in biofluids, where its concentration is influenced by norepinephrine’s synthesis, effects, and receptorial pathways. In addition, NGF levels may vary in different circadian phases. As several studies (Govoni et al., 2011) have highlighted, activation of various receptors can modulate NGF expression. For example, norepinephrine binding to beta-adrenoreceptors in cardiac stellate ganglia mediates upregulation of NGF synthesis (Hoard et al., 2008), while alpha sympathetic receptor activation decreases NGF release. Therefore, the rate of stimulus, its impulse, and the different context in which it occurs (in physiological and pathological conditions) may modify the neurotrophic output (Kreusser et al., 2008).

Other studies have demonstrated a sophisticated crosstalk between endothelin-1 (ET-1) (Ieda et al., 2004) and NGF: endothelin has been shown to enhance NGF levels in cultured cardiomyocytes (Cosgaya and Aranda, 1995). Therefore, endothelin-1 is also a pivotal molecule to ensure autonomic cardiac innervation homeostasis and an attractive molecular target in cardiac injuries.

## 4 Cardiac sympathetic innervation and risk of ventricular arrhythmia in myocardial infarction and heart failure

**FIGURE 1:**
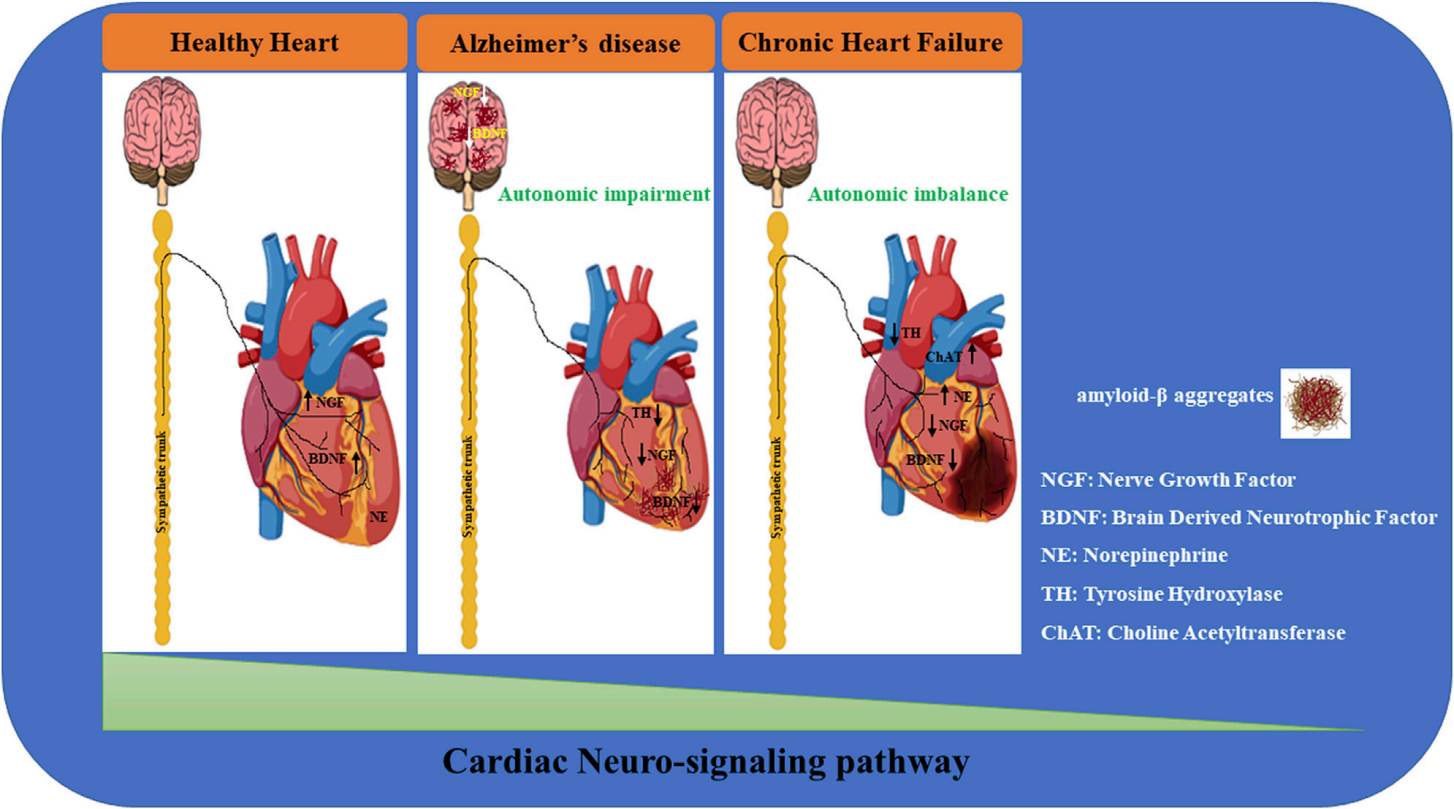
Neuro-signaling pathway dysregulation in relation to the development of heart damage. The gradual loss of neurotrophic factors (i.e.; NGF, BDNF) severely affects the neuro-signaling pathway of the brain/heart axis, increasing the incidence to develop cardiovascular disorders of different etiology. In Alzheimer’s disease (AD), amyloid-β accumulation significantly impacts the expression of neurotrophins, resulting in a maladaptive remodeling of the neuro-signaling pathway with a dramatic cognitive decline and dementia development. It is reasonable to hypothesize that the progressive degeneration of the neuro signaling pathway associated with neuronal impairment observed in the brain of AD patients may trigger a gradual decline in neurotrophic factors circulating levels, leading to a severe derangement of the cardiac nervous system, culminating in lethal heart disorders (such as AF, MI, HF). During heart failure, progressively, the blood pressure drops together with the cardiac output, resulting in brain hypoperfusion, thus leading to sympathetic nervous system hyperactivation. This induces norepinephrine (NE) over-release from sympathetic fibers, culminating in the upregulation of catecholamine circulating levels. In parallel, neuro-signaling is downregulated in the myocardium (green triangle), with progressive impoverishment of autonomic nerve fibers, leading to extended myocardial denervation.

Cardiovascular disorders represent the predominant cause of death worldwide, together with metabolic alterations and neurodegenerative disorders such as Alzheimer’s disease. Clinical studies show that a cardiac nervous system (Wake and Brack, 2016) derangement may be the underlying causal factor in different heart diseases: the dysfunction of the cardiac innervation gradient produces a cardiac performance impairment with consequential nerve fiber abnormalities, responsible for the arrhythmogenic phenomenon (Franciosi et al., 2017) described in different cardiovascular diseases, in particular atrial fibrillation, ventricular arrhythmia, myocardial infarction, and heart failure (Hunt et al., 2001) (Figure 1).

### 4.1 Atrial fibrillation

Atrial fibrillation (AF) (Miyasaka et al., 2006) is the most common cardiac electrical disorder, with its prevalence increasing in recent decades, as shown in epidemiological data from the Framingham Heart study (Benjamin et al., 1994; Kornej et al., 2022). Cardiovascular aging contributes to the development of arrhythmia; thus, its prevalence is age-related: 70% of AF patients are over 65 years old. Recently, an increase in this condition has been documented in the occidental population, due to both increased aging and multifactorial genesis. The increasing prevalence of atrial fibrillation has been associated with relevant social, economic, and healthcare policy implications. AF shows a rapid and chaotic electrical atrial activity (400–600 bpm) leading to inadequate mechanical cardiac performance.

The diagnosis occurs with electrocardiography analysis detecting the loss of “P” waves on the isoelectric line and the irregularity of R- R cycles. A specific classification of AF (Benjamin et al., 1994; Gallagher and Camm, 1998; Kornej et al., 2022), based on duration and interruption of the sustained arrhythmia, identifies several patterns.

- paroxysmal atrial fibrillation (under 7 days within spontaneous arrest, usually in the first 48 h) (Yalcin et al., 2015);
- persistent AF, which requires a pharmacological or electrical approach for its arrest, usually longer than 7 days.
- long-standing persistent AF, lasting more than 1 year when adopting a rhythm control strategy.
- permanent AF (Gould et al., 2006), when it does not respond to specific antiarrhythmic drugs or electrical cardio-reversing strategy.

The electrical dysfunction is characterized by a “storming electrical impulse” with alteration of cardiac rhythm (Wyse et al., 2002; Cappato et al., 2005; Gould et al., 2006; Miyasaka et al., 2006; de Chillou et al., 2008; Roy et al., 2008) and performances; AF physiopathology is multifactorial: alcohol abuse, hyperthyroidism, mechanical and phlogistic stimulation may accelerate the arrhythmogenic process upon critical atrial morphology. Alterations in conduction speed and reduction of the refractory period are favoured by arrhythmogenic substrates (Nguyen et al., 2009) to promote a microcircuits outflow that may preferably clash, burn, merge and lapse upon fibrotic and hypertrophic tissue, due to histopathologic remodeling. Specifically, as demonstrated, the ANS contributes to both the onset and progression of this disease (Coumel et al., 1978). Of note, the structural basis for this theory derived from the discovery of sizeable autonomic innervation fibers density and ganglionated Plexi (GP) associated with the pulmonary veins (PV) and within the atrial tissue, from which AF triggers often originate (Haïssaguerre et al., 1998). However, to better understand the etiology of AF a lot of studies sought to better characterize cellular substrates of AF. Specifically, the high density of β-adrenergic receptors and M2-type muscarinic receptors (cholinergic) in atrial and PV myocytes suggested these cells may directly communicate with the ICNS (Löffelholz and Pappano, 1985; Alexander et al., 2008). Yet, due to numerous factors (e.g., age and host environment), cardiomyocytes may respond differently to the same neural stimulus (Boknik et al., 2009). Part of this heterogeneity at the cellular level may be due to atrial remodeling that culminates in atrial fibrosis and alterations in cell signaling, such as calcium handling (Díaz et al., 2004; Hove-Madsen et al., 2004). Additionally, a sub-population of specialized cells, with their own pacemaker activity, was identified in the human atrium, pulmonary vein-left atrial (PV-LA) junction, and PV tissue (Cheung, 1981; Masani, 1986). These nodal-like cells interact with neurons from the ICNS and exhibit histological features of cardiac pacemaker similar to sinus node cells (Masani, 1986; Airaksinen et al., 1999). Accordingly, local innervation-induced modifications in the atrial myocardium’s electrophysiological environment may act as a stimulus for the further progression of sustained AF.

More recent electrophysiological reports (Wickramasinghe and Patel, 2013) and therapeutic results (de Chillou et al., 2008; Wickramasinghe and Patel, 2013; Chen et al., 2014) obtained with electrical ablation (Cappato et al., 2005) have suggested, as a new hypothesis, that a focal genesis, the “vagal or sympathetic trigger”, plays a key role in AF.

The persistence of AF induces cellular and subcellular modifications (such as intracellular Ca^2+^ accumulation, sarcolemma reduction, and glycogen accumulation), responsible for atrial physiological and structural abnormalities.

The atrial innervation is modulated by a sophisticated crosstalk between the two limbs of the cardiac autonomic nervous system (Jamali et al., 2017), whose imbalance causes the hyperfiring of cholinergic fibers, that support the vagotonic AF, and adrenergic axons that sustain the sympathetic AF (Li et al., 2013). Some authors have demonstrated in animal models that simultaneous choline-adrenergic activation easily induces AF. The arrhythmogenesis phenomenon can be driven by strong adrenergic waves, produced by stress conditions such as exercise and emotional changes. Patients suffering from “vagal trigger” AF are mostly healthy individuals with no evidence of cardiac disease. Conversely, patients with “sympatotonic” AF are usually elderly with different comorbidities and structural heart frailty.

Indeed, many clinical and preclinical reports have demonstrated cholinergic and sympathetic events to be triggers of AF. Evidence in canine models (Swissa et al., 2005; Tan et al., 2008) showed how simultaneous vagal and adrenergic stimulation (Ng et al., 2001) represents the main stimulus to ectopic focus activation. Other authors have demonstrated that silencing of paroxysmal AF (Yalcin et al., 2015) is triggered by rapid atrial spread, through bilateral cardiac ganglia (Xie et al., 2011) and cardiac vagal limbs ablation, producing total sympathovagal excitement abolition.

Neural modifications (Saygili et al., 2012), prompted by rapid atrial spread, also suggest atrial adrenergic fibers rejuvenation (Kimura et al., 2007). Sympathetic hyperinnervation, driven by norepinephrine spillover through the β–adrenergic stimulation (Suita et al., 2015), promotes the increase in protein kinase A (PKA) activity, modulated by ryanodine receptors (RyR2) hyperphosphorylation on atrial cardiomyocytes. The calcium released by atrial RyR2 induces fibrosis and apoptosis of myocytes and promotes the morphological transformation of atria, which in turn sustains the AF propensity.

### 4.2 Myocardial infarction

MI is a common cardiovascular disease; its incidence runs in parallel with age and lifestyle, showing a spike in patients over 75 years old. Many risk factors are related to MI: smoking, hypercaloric diet, dyslipidemia, sedentary life, alcohol abuse, high blood pressure, and others. MI mostly occurs as a consequence of coronary artery disease and coronary stenosis and results in hypo-perfusion of the heart and cardiac oxygen deficiencies in specific cardiac areas, identified as ischemic territory. The main MI symptoms are chest pain or discomfort, which may radiate to the shoulder, arm, back, neck, or jaw, shortness of breath, nausea, faint, cold sweat, or fatigue, with women more exposed than men to develop atypical signs. These macroscopic abnormalities (Davis et al., 1988) also translate into molecular disorders driven by NGF. Indeed, NGF levels extensively increase after MI (whereas its expression decreases during heart failure and ventricular injury), triggering the sprouting of neurites and neuronal modifications (Davis et al., 1988; Hassankhani et al., 1995; Cao et al., 2000a; Zhou et al., 2004; Oh et al., 2006; Lorgis et al., 2009). MI is characterized by a continuous and sustained catecholaminergic tone, to contrast the decreased irroration of the ischemic area. This effect determines beta-adrenoreceptors overstimulation by NE spillover (Hasking et al., 1986) with an upregulation of NGF, which promotes fiber remodeling and cardiac hyperinnervation (Li and Li, 2015), which is eventually responsible for the development of cardiac arrhythmia. A sinusoidal trend in NGF expression has been demonstrated during MI by different scientific reports (Zhou et al., 2004). As observed in murine models, the first hours after MI with beta adrenoreceptors activation are characterized by NGF overproduction, while after the development of the consequential cardiac cell death, a decline in NGF levels is gradually established, inducing heart swelling due to alpha-adrenoreceptors stimulation (Kimura et al., 2010). Myocardial infarction is characterized by an increase in sympathetic output with a progressive reduction of parasympathetic activity centrally, which could represent a potential therapeutic target to improve cardiac function (Airaksinen et al., 1999; Olshansky et al., 2008; Ardell et al., 2016; Shivkumar et al., 2016). Specifically, cholinergic nervous system remodeling with slight oscillations in acetylcholine expression was observed in the apex, anterior wall, and lateral wall of the LV in healthy pigs compared to the MI experimental group (Vaseghi et al., 2017), accompanied by significant impairment in basal function and input of the cholinergic nervous system within the ICNS (Vaseghi et al., 2017). Moreover, morphological remodeling in the nodose ganglia was documented in the MI model, specifically characterized by a significant upregulation in calcitonin gene-related peptide (CGRP) and TH-positive neuronal cells, in parallel with a gradual decline in neuronal nitric oxide synthase levels accompanied by relevant neuron size increase (Ardell et al., 2016; Salavatian et al., 2017). These results indicate that intra and extracardiac cholinergic maladaptive remodeling develops in the myocardial ischemic injury context, with intact cholinergic nervous pathways but decreased cholinergic input (Vaseghi et al., 2017). These intact parasympathetic pathways may represent a novel therapeutic target for the treatment of chronic cardiovascular disorders, through VNS.

### 4.3 Neuromodulation in MI

Impaired sensory information is one of the main drivers of acute autonomic responses to ischemic injuries and the progression of cardiac disorder. Wang et al. described an attenuation of HF in cardiac post-ischemic experimental models through selective chemical ablation of cardiac transient receptor potential cation channel subfamily V member 1 (TRPV1) receptors using epicardial resiniferatoxin (Wang et al., 2014). Thus, tailored neuromodulation may potentially affect sensory transduction in a reversible and on-demand manner (Wang et al., 2014; Ardell et al., 2016; Salavatian et al., 2017). Temporary occlusion of the left anterior descending (LAD) coronary artery results in nodose hyperactivity. However, direct (cervical vagal, VNS) and remote (thoracic the spinal cord, SCS) neuromodulation alleviates nodose response to transient myocardial ischemia events induced by repeated LAD ligations (Salavatian et al., 2017). Together, this evidence suggests that preventive neuromodulation affects myocytes metabolism, thus improving their resistance to stress stimuli (McGuirt et al., 1997; Southerland et al., 2007; Wang et al., 2014; Salavatian et al., 2016). Many experimental models have shown a strong infiltration of macrophages in the ischemic area. Immune cells contribute to increasing NGF production, activating its receptors TrkA and p75 (Ben-Zvi et al., 2007; Carter et al., 2010), which are also expressed in endothelial cell bodies. The increased release of NGF triggers a further hyperinnervation modulated by TrkA binding, whereas p75 (Lee et al., 1994; Habecker et al., 2008) activation induces the release of TNF alpha, a potent inflammatory modulator, which induces cardiomyocytes apoptosis. As emerged from different clinical data, the risks to develop severe arrhythmias (ventricular tachyarrhythmia, ventricular fibrillation) is higher for 30 days after MI and NGF sympathetic hyper-sprouting is the main promoter of arrhythmias and ventricular dysfunction (Hu et al., 2014a; Pius-Sadowska and Machaliński, 2021). During the inflammatory response, myocardial cells secrete phlogistic hypertrophic factors (Matsui et al., 1996), such as cardiotrophin-1 (CT-1), ciliar neurotrophic factor (CNTF), and leukemia inhibitory factor (LIF) (Sun and Zigmond, 1996), which are capable of both abolishing NE release and inducing cholinergic marker (choline acetyltransferase -ChAT and vesicular acetylcholine transporter- VaChT) synthesis in cardiac stellate ganglia (Zhou et al., 2005), via the interleukine-6 (IL-6) glycoprotein 130 (gp130) pathway. This process promotes a phenotype transdifferentiation. Indeed, immunolabelling showed cholinergic staining of adrenergic stellate neurons, with coexpression of TH and ACh immunopositive fibers (Olivas et al., 2016). Moreover, adrenergic fiber depletion occurs. All the reported changes are connected to NE overproduction, which suppresses TH synthesis (through proteasomal degradation) (Shi and Habecker, 2012) and attenuates norepinephrine reuptake. As a result, a decrease in NE cardiac expression is observed, accompanied by a reduction in MIBG reuptake (Dae et al., 1991). Experimental studies on mice (Rana et al., 2009) have shown that ChAT (key enzyme for acetylcholine synthesis) gene silencing (mice ChAT/- knockout) in adrenergic neurons reduced myocardial infarction. Specifically, Olivas and others found that the deletion of ChAT from sympathetic neurons abolished the post-MI increase in cardiac ACh content, culminating in ACh levels significantly lower than WT post-MI hearts. Conversely, NE levels did not change. This suggests that the increased ACh observed in the left ventricle after MI was due to the production of ACh by sympathetic nerves. Notably, the transient increase in cardiac ACh amount coincided with increased expression of the genes required for ACh production in the stellate ganglion and the appearance of ChAT and VAChT protein in TH + cardiac sympathetic neurons. In summary, these data indicate that cardiac sympathetic nerves produce ACh in addition to NE after MI (Olivas et al., 2016).

The shift from adrenergic to parasympathetic phenotype is also sustained by glycoprotein 130 receptors that allow the “cholinergic dress acquisition” of adrenergic neurons. Supporting a transitory cholinergic trans-differentiation in adrenergic cardiac neurons, after gp130 receptor deletion, parasympathetic genes were not expressed post- MI. Thus, transient sympathetic co-secretion of norepinephrine and acetylcholine may favor a strong reaction mediated by inflammatory cytokines (Nian et al., 2004), inducing cardiac remodeling and lethal arrhythmias which may cause sudden cardiac death (Cao et al., 2000a; Chen et al., 2001). Although functional implications of the sympathovagal signal (Cao et al., 2000a; Chen et al., 2001; Olshansky et al., 2008; De Ferrari et al., 2011) remain uncertain, multiple reports (Emanueli et al., 2014) suggest that concurring actions of NE and ACh from sympathetic fibers may underlie heart impairment and arrhythmia propensity. NGF and inflammatory cytokines are identified as new important therapeutic targets on which we may act to prevent the dramatic cardiac remodeling processes post-MI and ventricular injuries. As demonstrated, the use of agonists or antagonists of the NGF receptor (TrkA) (Kuruvilla et al., 2004) or the NGF gene transport may improve angiogenesis and cardiomyocytes survival, thus promoting the cardiac response after a few weeks post-myocardial infarction (Meloni et al., 2010).

### 4.4 Heart failure

Neuronal remodeling reaches its final phase with the development of heart failure (Jessup and Brozena, 2003), a complex clinical syndrome also characterized by a complete impairment of the autonomic nervous system. HF incidence is clearly age-related: 0.8% of individuals between 50 and 59 years old and up to 9.2% of subjects between 80 and 89 years old suffer HF, with occurrence doubling for each decade beginning from 40 years of age. The increased risk for males in younger ages is inverted in advanced decades, with females presenting a higher risk after menopause. Age represents the main risk factor for heart failure development (Di Bari et al., 2004), which is mostly associated with acute and chronic MI, hypertension, and valvular disorders (in particular, degenerative aortic stenosis and mitral regurgitation). As emerged in many reports (Vasan et al., 1995), HF is characterized by morpho-structural changes, with cardiomyocytes apoptosis and interstitial fibrosis, associated with left ventricle concentric hypertrophy (Wilde et al., 2008), aortic stiffness, and increased susceptibility to myocardial ischemia. Different comorbidities and simultaneous disorders, such as diabetes mellitus (Faerman et al., 1977), atrial fibrillation, lung injuries connected to Chronic Obstructive Pulmonary Disease (COPD), and kidney function worsening, can promote HF syndrome.

The pathophysiological basis of HF is constituted by a blood pressure decrease, which triggers a “reaction flow”; the cardiac output lowering is detected by carotid sinus receptors (Wang et al., 1990) that send impulses to the vasomotor bulbar center; this process determines a “switch” and promotes a strong “adrenergic firing” on the myocardial tissue. Sympatho-excitation in heart failure is also associated with reduced sensitivity of sympathetic-inhibitory reflexes, such as the arterial baroreflex and the cardiopulmonary reflex, to an increase in activity of peripheral chemoreceptors at a high plasma concentration of angiotensin II and to the reduction in the synthesis of nitric oxide (Lataro et al., 2017). The above-mentioned mechanisms exert a compensatory effect to ensure suitable cardiac contractile strength for a while, but sympathetic (Zhang and Anderson, 2014) hyperactivity rapidly contributes to disease progression and reduction of adrenergic stimulation (Eisenhofer et al., 1996). Thus, from asymptomatic ventricular dysfunction, the process evolves to HF, with cardiac output decline and lung hypertension. Adrenergic and RAAS system hyperstimulation acts as a compensatory mechanism to assure cardiac perfusion, but with disease progression, increases cardiac workload and induces peripheral vasoconstriction. Thereby kidney and skeletal musculature ischemia are promoted, resulting in decreased exercise tolerance and renal dysfunction.

### 4.5 Neuromodulation in HF

The extensive activity of the endocrine system and adrenergic circuits promotes a bulky augmentation of phlogistic cytokines (IL-6; LIF; CT-1) (Ure and Campenot, 1994), secreted by stressed myocardial cells, resulting in myocytes apoptosis and metabolic cellular disorders, reactive oxygen species production and cardiac derangement. Naturally, in this contest the autonomic dysregulation also causes “peripherical and molecular turbulences”: adrenergic hyperfiring triggers a strong spillover of circulating catecholamines (especially norepinephrine) (Chidsey et al., 1965; Chidsey et al., 1963; Cohn et al., 1984; D'Elia et al., 2014), that exert their functions through beta-adrenoreceptors binding and try to contrast the lowering of blood pressure. In this phase, a robust hyperinnervation with NGF overproduction (Hassankhani et al., 1995) has been demonstrated to be mediated by beta-adrenoreceptors in different rodent experimental models with left anterior descending coronary artery ligation (D'Elia et al., 2014). A paradoxical phenomenon manifests during this process: hyperexcitement of sympathetic circuits tries to support the cardiac activity but, progressively, the inhibition of TH synthesis induces a decrease in norepinephrine cardiac levels with stimulation of alpha2-adrenoreceptor (alpha2-ARs), which also leads to reduction of NGF expression. Although the reduction of cardiac NE levels was assessed in an overt HF setting, the progressive accumulation of catecholamines in the synaptic space promotes the alpha2-ARs stimulation, that through the inhibitory presynaptic feedback loop tries to limit NE additional release. However, since the alpha2-ARs is coupled to Gi, the activation of this receptor contributes to a decline in NGF expression (Starke et al., 1975; Jie et al., 1987; Kubo et al., 1989; Grossman et al., 1991; Parker et al., 1995; Bylund, 2005; Govoni et al., 2011).

The entire process develops in a severe disruption of cardiac sympathetic fibers (Lorentz et al., 2013). Accordingly, autopsied human heart samples from patients with congestive heart failure (CHF) have revealed a dramatic loss of adrenergic TH-reactive fibers (Kaye et al., 2000; Qin et al., 2002).

From myocardial infarction to heart failure development, neurons in the adrenergic ganglia show an overlap between cholinergic and adrenergic markers and gradually promote the synthesis of parasympathetic modulators (Kanazawa et al., 2010), mediated by “cardiokines” (IL-6; LIF; CT- 1) secreted from “worn cardiomyocytes”. The mechanisms responsible for this switch still remain unclear, although it probably constitutes a protective mechanism instigated in the heart in order to avoid lethal consequences, such as arrhythmias, induced by chronic sustained adrenergic activity, and to help preserve innervations in the denervated areas.

### 4.6 Alzheimer’s disease, neurotrophins, and cardiovascular disease

AD is a complex neurodegenerative disorder and the main form of dementia, characterized by a progressive accumulation of cerebral parenchymal β-Amyloid (Aβ) plaques and intraneuronal tau neurofibrillary tangles, associated with widespread brain inflammatory processes. The disease induces a gradual cognitive decline with progressive memory dysfunction (Kanazawa et al., 2010; Castellani et al., 2010; Small et al., 1997-29). Although the main symptoms of AD predominantly concern the CNS, numerous extracerebral and systemic effects have been recently identified, which may also affect the cardiovascular tissue. In this regard, several studies have described interesting associations between Alzheimer’s disease and cardiac disorders, such as AF (Ihara and Washida, 2018), HF (Cermakova et al., 2015; Cohen and Mather, 2007; Hoth et al., 2008), and coronary artery disease (CAD) (Roger, 2013; Tublin et al., 2019). It was recently observed that AD patients exhibit Aβ deposits, besides cerebral areas, also in the cardiac tissue. Cardiac amyloid deposition was also found in patients affected by idiopathic dilated cardiomyopathy (iDCM) (Li et al., 2006b; Gianni et al., 2010; Troncone et al., 2016). However, nothing is currently known about the timeline of Aβ accumulation in the heart of AD patients and evidence is lacking in regard to the levels and concentrations of Aβ aggregates in the AD heart compared to the brain. It is however conceivable that similar pathological mechanisms, such as a failure of proper clearance of amyloid (including Aβ), may be responsible for both.

Age is the main risk factor for both AD and HF (Kivipelto et al., 2005; Viña and Lloret, 2010; Hölscher, 2011), and these disorders exhibit many other common risk factors, including metabolic and cardiovascular disorders, diet, lifestyle, or the higher risk in females after menopause. Importantly, many recent clinical studies demonstrated an interesting interaction between cerebral hypoperfusion and cognitive dysfunction in HF patients, strengthening the links between cardiovascular conditions, vascular dementia, and AD (Mansur et al., 2018; Ventoulis et al., 2021; Li et al., 2022). Notably, during HF, blood pressure progressively drops, resulting in brain hypoperfusion with a remarkable oxygen loss, increase in oxidative stress and neuronal metabolic impairment (Moreira et al., 2005; Pirchl and Humpel, 2009; Cermakova et al., 2015). Interestingly, clinical investigations demonstrated a positive correlation between cognitive deterioration and HF severity (Cohen and Mather, 2007-; Hoth et al., 2008). Neuroimaging studies corroborated these findings, describing significant brain morphological changes in HF patients (Vogels et al., 2007; Cermakova et al., 2015). Specifically, Kumar et al. observed severe demyelination in HF patients, associated with a dramatic cerebral atrophy that induces neuronal maladaptive remodeling with consequential impairment of axonal circuit functionality (Vogels et al., 2007; Kumar et al., 2011; Cermakova et al., 2015). Yet, the neuronal deterioration found in HF might be connected to a more complex etiopathology. Indeed, we and other groups propose that HF itself may also be a result of cardiac Aβ accumulation triggered by AD pathology, which would instigate an accumulation of multiple amyloid proteins in an understudied potential vicious cycle between brain and heart deterioration (Troncone et al., 2016; Wang et al., 2017; Sanna et al., 2019). However, more studies are needed to demonstrate this hypothesis.

Li and others have identified interesting missense mutations in PSEN1 (Asp333Gly) and PSEN2 (Ser130Leu) genes observed in AD patients affected by HF (Li et al., 2006b). In line with this, the Del Monte group has described the same AD-related gene variations in iDCM patients that exhibited cardiac inclusions consistent with amyloid accumulations (Gianni et al., 2010). All these findings provide evidence of the strong existing correlation between Alzheimer’s disease and heart failure conditions that likely may share common, yet poorly investigated, pathophysiological mechanisms. For instance, it is well known in the AD field that Aβ oligomers promote significant neurotoxicity, and among other toxic effects, they affect calcium currents, thus impairing neuronal homeostasis, synaptic health, and neurotransmission. Interestingly, similar detrimental effects on calcium handling were detected in cardiomyocytes’ challenged with Aβ oligomers, thus suggesting similar mechanism triggered by Aβ in the neuronal and cardiovascular environment (Gianni et al., 2010; Jang et al., 2022).

Besides synaptic, neuroimmune, and vascular dysfunction, AD is accompanied by a significant impairment in neurotrophic signalling, with a progressive loss of the two main neuromodulators, NGF and BDNF (Allen et al., 2011). Specifically, both these neurotrophins are severely affected during the neurodegenerative process in the early stages of the disorder. Indeed, several studies have described detrimental effects mediated by Aβ on the expression of neurotrophins, resulting in cognitive decline (Garzon et al., 2002; Arancio and Chao, 2007). Notably, a gradual failure of NGF signaling, with progressive TrkA (NGF receptor) downregulation was shown in the cholinergic basal forebrain nuclei (ChBF) (Davies and Maloney, 1976; Counts and Mufson, 2005), accompanied by a severe Aβ-induced BDNF decline in the cortex and hippocampus (Arancio and Chao, 2007). These progressive alterations in the neurotrophic pathway are associated with a gradual cognitive impairment both in elderly patients and in aging murine models (Tessarollo and Hempstead, 1998; Donovan et al., 2000; Sarter and Bruno, 2004; Jansen et al., 2007; Scharfman and Chao, 2013; Anastasia et al., 2014). Therefore, it is reasonable to hypothesize that restoring the neurotrophic pathway via viral vectors that produce NGF and BDNF, or through neurotrophins receptor agonists, may counteract neuronal deterioration and attenuate synaptic degeneration, thus improving cognitive decline, as proposed by some clinical studies (Graham et al., 2017; Castle et al., 2020; Ruiz-González et al., 2021; Eyjolfsdottir et al., 2022). Curiously, recent studies have also evidenced a significant dysregulation of the BDNF signaling pathway during aging (Miranda et al., 2019; Molinari et al., 2020). Elia and colleagues have described severe impairment in the cardiac autonomic nervous system exhibited by aged rats, associated with a significant deterioration of both adrenergic and cholinergic nerve fibers, together with a relevant downregulation in cardiac BDNF protein levels (Elia et al., 2021). This autonomic deregulation may be, at least in part, responsible for the risk of developing cardiovascular disease in the aging/Alzheimer’s heart. Accordingly, we speculate that a progressive degeneration of the neurotrophic pathway in AD may trigger a gradual decline in circulating NTFs levels, also resulting in a derangement of the cardiac nervous system, culminating in lethal heart disorders (such as AF, MI, HF). Therefore, neurotrophic factors may represent innovative and promising therapeutic frontiers to improve cerebral and cardiac prognosis and ameliorate the quality of life for both AD and HF patients.

In line with our hypothesis of a link between heart and brain dysfunction, recent clinical investigations, as well as studies in animal models, have highlighted the beneficial effects promoted by different cardioprotective drugs (i.e., Beta-blockers, Angiotensin Converting Enzyme (ACE) inhibitors, sartans, and Aldosterone receptor antagonists) in Alzheimer’s disease and cognitive dementia. The cardioprotective drugs, in addition to providing preventive effects (Carey and Fossati, 2022), also appear to prevent the neuronal accumulation of Aβ aggregates and result in a significant improvement in memory impairment in some clinical studies as well as in multiple animal models (Chen et al., 2020; Lee et al., 2020; Drews et al., 2021; Ouk et al., 2021; Wang et al., 2021; Beaman et al., 2022; Deng et al., 2022; Mehdipour et al., 2022). This evidence points to the possibility that combination therapies containing these compounds may have efficacy for the treatment and prevention of both cardiac diseases and dementia in older adults.

## 5 Proposed therapies to reverse the remodeling of cardiac innervation: Functional and molecular aspects

The sustained adrenergic hyperactivity described in post-MI models represents a compensatory organic strategy to counteract the blood pressure reduction. However, this initial mechanism only represents a transitory solution, because the continuous and robust sympathetic response leads to congestive heart failure (Rich et al., 1995). During heart failure development, adrenergic hyperactivity (Leenen, 2007) is supported by RAAS activation; in addition, a strong neuroendocrine stimulation mediated by different agents can be detected: norepinephrine, renin, angiotensin, and aldosterone activate a “stimula tempest” which induces stress and cardiac loss of function. This “storm” also stimulates nerve fiber remodeling and changes in cardiac innervation.

The higher NE circulating levels encourage β-ARs binding and the consequent cardiac fibers hypersprouting, which results in remodeling of the cardiac autonomic system, the main recognized cause of ventricular hyperinnervation and tachyarrhythmia (Shen and Zipes, 2014). Concurrently, the neuroendocrine pathway (Carlson and Wyss, 2008) promotes the production of cytokines by damaged myocytes, accelerating metabolic cellular disorders, ROS production and myocardial cell apoptosis (Caporali et al., 2008).

In this context, our Review analyzes current therapies to prevent and neutralize heart failure and their possible roles in reverting the cardiac neuronal maladaptive remodeling (Bristow et al., 2004).

### 5.1 Beta-blockers

#### 5.1.1 Animal studies

The roles of beta-blocker therapy on cardiac sympathetic remodeling have been observed also in animal models. Clarke et al. (2010) speculated tight beta adrenoreceptors autofeedback in a murine model of heart failure, induced by myocardial infarction. Notably, via immunohistochemical and molecular analysis, they found a beta-1 presynaptic auto-adrenoreceptor in nerve fibers, most likely associated with G-inhibitor proteins, which modulate axonal sprouting. The authors postulated that the β-adrenergic signaling pathway is involved in the restoration of nerve fibers after heart failure. Other scientific studies (Fagerberg, 2000) support the mentioned mechanism. Reports in rabbits have described that the strong fiber hypersprouting in the border area of myocardial infarction is reduced by metoprolol therapy (Cao et al., 2000b). Further investigations may be useful to establish if the β-ARs inhibitors’ effect on hyperproliferation of cardiac fibers after MI would also be able to alter the autonomic system of the human heart. Wang and others have demonstrated that metoprolol improves cardiac fiber sprouting in MI rabbits with heart failure (Jiang et al., 2007; Wang et al., 2013). Notably, the authors have evaluated a decrease in different phlogistic markers: IL-1β, TNF-α, and NF-κB, associated with NGF (Crowley et al., 1994) decline and upregulation of the α-inhibitor factor of NF-κB (IκBα). These data support the hypothesis that β-1 ARs selective inhibitors reduce the exaggerated cardiac innervation, preventing ventricular remodeling and myocardial injuries, partially by counteracting cardiomyocytes apoptosis mediated by the inflammatory response.

The inflammatory process developed during MI and extended in HF involves different cytokines, especially the powerful peptide angiotensin- II, produced following the RAAS (Huang and Leenen, 2009) circuit derangement. HF implies a massive release of AT- II and catecholamines (especially NE), which promote autonomic cardiac system impairment, worsening the pathological framework of HF.

#### 5.1.2 Human studies

ESC Guidelines identify beta-blockers as the first pharmacological solution to contrast HF (Floras, 2009) and the related complications (Kramer et al., 1999). Beta-blockers restore the signaling of beta sympathetic receptors in cardiac cells, “extinguished” by over-release of catecholamines in HF. Furthermore, they are also antiarrhythmic drugs for their negative chronotopic effect (Feldman et al., 2010), which reduces the heart rate and slows down atrioventricular pacing. In addition, they play a role in the reduction of myocardial contractility (negative inotropic effect), resulting in a cardiac output decrease, and acting as antihypertensive drugs. In particular, β-ARs antagonists improve myocardial oxygen use and ameliorate the ventricular filling (Lucia et al., 2018). However, caution is needed, in particular in elderly patients, in order to avoid adverse effects (such as bradycardia and hypotension). Importantly, clinical trials such as CIBIS II (Cardiac Insufficiency Bisoprolol Study II) (Krum et al., 2011a), COPERNICUS (Carvedilol Prospective Randomized Cumulative Survival) (Cheng and Nayar, 2009), MERIT-HF (Metoprolol CR/XL Randomized Intervention Trial in Congestive Heart Failure) (Fowler, 2004), have demonstrated the efficacy of beta-ARs antagonists in Chronic Heart Failure (CHF), reporting a significant decrease in mortality (34%), as well as a reduction in hospitalization from 28% to 36%.

Many studies (de Peuter et al., 2013) analyzed the efficacy of beta-blockers in reverting the cardiac innervation remodeling as monotherapy or in synergistic effect with other cardioactive drugs, such as ACE inhibitors, sartans, and aldosterone receptor antagonists. In a small study on 36 patients with HF, Kubo et colleagues (Kubo et al., 2011) have demonstrated that extended treatment with a selective (metoprolol) or not selective (carvedilol) beta antagonist restored adrenergic axonal cardiac reflexes, promoting the recovery of signal propagation after the pharmacological therapy. There were no differences in treatment effects between the two beta-blockers, suggesting that metoprolol utilization alone may also reestablish the heart’s normal autonomic activity. ACE inhibitors also contributed to improving the positive effect of β-ARs antagonists in cardiac disorders such as heart failure and sudden cardiac death (Fukuda et al., 2015). The efficacy of beta-blockers also manifests in norepinephrine cardiac terminal fibers reuptake, as shown in different reports, which described the efficacy of carvedilol as a non-selective beta-adrenergic receptor antagonist. Chizzola and collaborators (Chizzola et al., 2006) have investigated the effectiveness of carvedilol to ameliorate the prognosis of patients with heart failure (Jacobson et al., 2010). The authors reported an improvement in MIBG reuptake in 53 patients with HF that received 42 mg/day of carvedilol (a higher dosage than in other studies). This reflected a restoration of the cardiac autonomic system accompanied by left ventricular performance improvement [diastolic filling, ejection fraction (EF)].

Patients with HF on beta-inhibitors treatment have shown a reverse in ventricular derangement, as shown by an improvement of cardiac MIBG re-uptake (Merlet et al., 1999; Toyama et al., 2003). Despite massive sympathetic axon proliferation in the proximal ischemic area (Cohen-Solal et al., 2005; Hasan et al., 2006; Wernli et al., 2009), beta-blockers did not further increase the cardiac innervation, suggesting a probable local autoregulation mechanism. Despite the improvement in hospitalization ratio and survival of HF patients, further investigations are still needed to clarify the efficacy of carvedilol in the neutralization of autonomic remodeling of the heart.

### 5.2 ACE inhibitors/sartans

Angiotensin Converting Enzyme inhibitors reduce the activity of the ACE, which is responsible for the conversion of Angiotensin- I (AT- I) -produced as a result of different reactions between a liver compound (i.e., Angiotensinogen) and a kidney hormone (i.e., renin)- in the peptidic marker AT- II. Therefore, ACE inhibitors stop the inflammatory consequences and decrease norepinephrine, inducing remodeling of the cardiac fibers network and restoring cardiac performance. Moreover, RAAS dysfunction induces hyperproduction of the cortical adrenal mineral corticoid hormone “aldosterone”, whose function is to retain sodium in organic fluids, increasing the systolic ventricular tension and thereby causing hypertension. ACE inhibitors represent an important pharmacological treatment for cardiac output hypertension. Similarly, angiotensin receptor antagonists (ATRs), also called sartans, play a crucial role in the management of hypertension and HF (Cleland et al., 2005). These drugs counteract the adverse effects of AT- II, by blocking its presynaptic and/or postsynaptic receptors (Schlicker and Feuerstein, 2017) and restore RAAS signaling. Many scientific reports have explored the efficacy of ACE inhibitors, and their possible synergistic effect with sartans, both in human and animal models.

#### 5.2.1 Animal studies

Hardwick and colleagues have studied the implications of the ACE inhibitor captopril on cardiac plexus remodeling triggered during MI in a guinea pig model (Hardwick et al., 2015). Notably, they tested the involvement of AT1 receptor antagonists, such as losartan, together with AT2 receptor stimulation. Their data points to the importance of a balance between AT- II receptors, AT1Rs and AT2Rs. MI induced an increase both in adrenergic system activity, with NE spillover, and in angiotensin II-mediated cardiac hyperstimulation. Of note, captopril treatment did not modulate catecholamines levels or AT- II effects. Since other peptidases may produce AT- II, this mechanism might explain the failure of captopril to prevent AT- II-mediated cardiac disorders. Interestingly, losartan reverted the AT1Rs dysregulation due to the systemic AT- II upregulation that occurred during MI. Moreover, the authors observed a decrease in norepinephrine effects following the activation of post-synaptic AT- II receptors (AT2Rs), resulting in AT2Rs restoration and improvement of the cardiac autonomic circuit, which is impaired by MI. Thus, these results have highlighted the importance of receptor homeostasis between AT1Rs and AT2Rs for the cardiac autonomic modulation. Nevertheless, there are discrepant results from other studies about ACE inhibitors and sartans on cardiac remodeling. Indeed, Murakami and collaborators have described a cardiac output stimulation effect of captopril in a canine model, as well as the lack of effect on ventricular architecture and myocardial contractile endurance after irbesartan treatment of HF in rabbit models (Murakami et al., 1995; Murakami et al., 1996). Other research has shown that pigs have no improvement in ventricular chamber enlargement and its kinetics after therapy with the AT1Rs valsartan (Zhang et al., 2014). Similar evidence emerged from studies on rabbits in which valsartan has not shown any reduction in ventricular collagen deposition promoted by HF. In rat models, however, treatment with losartan exerted a decrease in diastolic blood pressure with an inadequate effect on the modification of the myocardium, while the ACE inhibitor quinapril promoted protective effects on heart performance, reducing stress after ventricular injuries. Kawai et al. (2000) have investigated the conjugated effect of quinapril and losartan on the recovery of cardiac functions in a rabbit model of HF. Interestingly, the data collected demonstrate ventricular geometry improvement by restoring NE reuptake from cardiomyocytes, through neutralization of the AT- II deleterious cardiac effects and enhancement of cardiac synaptic plasticity. Furthermore, the drug combination promoted beneficial activity against ventricular remodeling, thus arresting disease progression. These results underline the significant impact of a combined therapy with an ACE inhibitor and an AT1Rs antagonist on ventricular abnormalities.

#### 5.2.2 Human studies

Kasama et al. (2006) have examined 27 patients with congestive heart failure, treated with enalapril (ACE inhibitor agent) and/or valsartan (angiotensin receptor-1 blocker). The authors have reported that, after 6 months of pharmacological therapy, an increase in ventricular hemodynamic outcomes was detected via echocardiography analysis. Moreover, as evaluated through MIBG, which allows assessment of heart remodeling, the patients receiving therapy with valsartan showed a recovery of sympathetic activity. The comparative study between enalapril and valsartan highlighted the key role of sartans in HF treatment. This class of drugs may promote substantial advantageous effects on cardiac performance, by enhancing dynamic and structural cardiac physiology (ejection fraction, telediastolic volume) and restoring the nervous circuits of the heart (NE and AT- II cardiac levels reduction, improvements of nerve connections). To further support these data, other clinical studies have demonstrated a remarkable effect of ACE-antagonists and AT1Rs blockers in avoiding ventricular worsening (Tsutamoto et al., 2008).

### 5.3 Aldosterone and renin antagonists

Renin inhibitor agents, such as aliskiren, represent another important resource in HF treatment: acting on the RAAS system, this drug prevents the conversion of angiotensinogen to angiotensin I.

#### 5.3.1 Animal studies

In a rat model, Jia et al. (2013) observed a decrease in cardiac and circulating catecholamine concentrations and an improvement in cardiac nerve fibers, with a decrease in TH-positive fibers, in animals treated with aliskiren. In addition, a refractory period extension was detected, resulting in the prevention of severe ventricular arrhythmias (Cao et al., 2000b) linked to SCD (Solomon et al., 2005). By abolishing angiotensin- II production, a natural consequence of aliskiren administration is also the attenuation of the inflammatory effects. Thus, as other pharmacological classes (ACE inhibitors, sartans, and beta-1 antagonists) (Hardwick et al., 2012), aliskiren and similar agents can also reduce the ventricular susceptibility to cardiac rhythmic oscillations, suppressing the spread of the arrhythmogenic phenomenon and reversing the cardiac remodeling. Cardiomyocytes’ apoptosis with consequential myocardial injuries is also mediated by aldosterone, the main mineralocorticoid hormone released by the glomerular area of the adrenal cortex. This lipidic hormone exerts an antidiuretic effect, favoring the increase in blood pressure and the overload of the heart. Aldosterone-induced fibrosis of the cardiovascular tissue is also connected with RAAS remodeling. (Cannavo et al., 2019a). Hence, aldosterone receptor antagonists, such as spironolactone, also offer beneficial effects on cardiac remodeling (Cannavo et al., 2018; Cannavo et al., 2019b).

#### 5.3.2 Human studies

Kasama and others have analyzed the contribution of spironolactone on the recovery of cardiac performance after heart injuries, in a heterogeneous population of 30 patients affected by HF (Kasama et al., 2002). They observed a restoration in left ventricular features: diastolic filling pressure, relaxation of internal walls with improvement in systolic contraction strength and reduced myocardial tissue hypertrophy with a decrease in cardiac mass and restoration of the cardiac autonomic system, leading to an improvement in the clinical New York Heart Association (NYHA) functional class.

Another study (Herring, 2015), has pointed out the involvement of neuropeptide Y, that might favor the ventricular axonal network dysregulation and the onset of arrhythmias. Besides catecholamines, high plasma levels of NY have been detected in HF. This may support the adrenergic hyper tone via parasympathetic inhibition of cardiac ganglia, linking to its receptors Y-1 (identified both in human and rat heart) and triggering ventricular fibrillation, despite the administration of beta-blockers. Conversely, Y-2 receptors can control heart rate activating vagal control. The author proposes to introduce neuropeptide Y-1 receptor antagonists and agonists of Y-2 to prevent cardiac comorbidity and mortality in the canonical pharmacological treatment of HF.

### 5.4 Neuromodulation of the ANS in cardiovascular diseases

**FIGURE 2:**
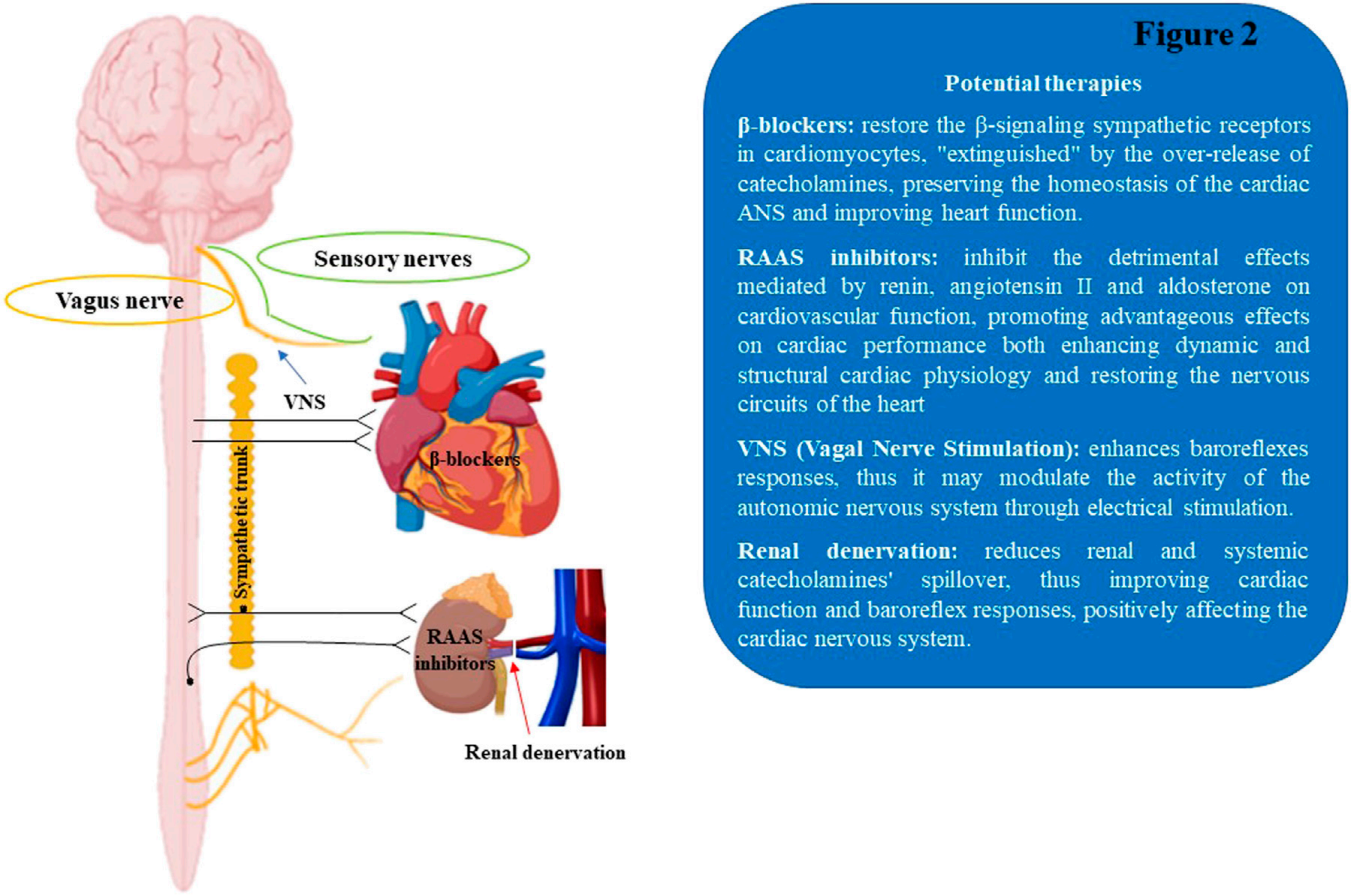
Schematic illustration of therapeutic approaches to modulate the cardiac autonomic nervous system. RAAS, renin-angiotensin aldosterone system; VNS, Vagal Nerve Stimulation.

The autonomic nervous system remodeling is one of the common factors involved in the etiopathological mechanisms of cardiovascular disorders, including cardiac arrhythmias, MI, and HF. Despite the advances in the medical and surgical fields, the progression of cardiac illnesses is still growing, together with the mortality rate (Florea and Cohn, 2014; McMurray et al., 2014; Shivkumar et al., 2016). Notably, the established pharmacological treatments for cardiac disorders act partially through neurohormonal inhibition, via β-ARs antagonism and/or RAAS blocking, thus modulating ANS activity, mitigating the aberrant cardiac electric events and restoring cardiac physiological homeostasis (Triposkiadis et al., 2009). As a matter of fact, the beneficial effect of the β-blocker therapy observed in HF patients is significantly proportionate to the degree of heart rate (HR) decrease (Fox et al., 2007). Specifically, cardiac prognosis improvement was obtained with treatments aimed at achieving low resting HR in heart failure, as demonstrated in the SHIFT (Systolic Heart Failure Treatment with the If Inhibitor Ivabradine) clinical trial, based on ivabradine therapy (Swedberg et al., 2010). Yet, numerous concerns remain regarding the neurohormonal modulation of the ANS tone through pharmacological approaches, including poor pharmacological specificity to discriminate the ANS branches (SNS and PNS) and considerable drug intolerance with recurring side effects. For instance, in a randomized controlled study conducted on select HF subjects (Lang et al., 1997; Cohn et al., 2003), the possible risks of moxonidine treatment emerged. This specific antihypertensive central medication decreased NE plasma levels, but did not show a selective block of sympathetic activity, thus increasing the morbidity and mortality risk in HF patients (Bristow, 2003). An extended knowledge of the dynamic connections between the nervous system and cardiac tissue has resulted in the development of novel therapeutic solutions based on the neuromodulatory stimulation of the ANS, including renal denervation and vagal nerve stimulation, which appear to exhibit significant beneficial effects in multiple cardiac disorders (Figure 2).

### 5.5 Renal denervation as an alternative solution for cardiovascular disorders

Adrenergic renal afferent and efferent fibers participate in the maintenance of cardiovascular homeostasis, counteracting stress stimuli that occur in cardiac injuries. Specifically, the renal sympathetic network is involved in the modulation of blood pressure, heart rate, and cardiac output (Esler, 2010). In chronic cardiac disorders (such as HF), the carotid chemoreceptors and baroreceptors transmit signals, such as those associated with decrease in blood pressure, cardiac output, and oxygen expenditure increase, to the brain, thus leading to RAAS activation, AT- II increase and in the central stimulation of sympathetic hyper tone (Murakami et al., 1997; Goldsmith, 2004; Li et al., 2006a; Paton et al., 2013). This culminates in renin over-release from renal glomeruli, resulting in a severe impairment of sodium and water handling (Ljungqvist and Wågermark, 1970; Goldsmith, 2004). Moreover, progressive renal hypoperfusion directly contributes to the SNS output centrally (Sobotka et al., 2011). This induces secretion of NE from adrenergic fibers, resulting in the upregulation of catecholamines circulating levels, which culminates in a dysregulation of the cardiac β-signaling pathway, thus fueling cardiac maladaptive remodeling and neurohormonal axis impairment (Böhm et al., 1988; Böhm et al., 1990). Interestingly, a long body of evidence demonstrates a tight correlation between cardiac and renal catecholamines spillover and morbidity/mortality rates (Hasking et al., 1986; Petersson et al., 2005). Therefore, multiple investigators proposed renal denervation as a therapeutic solution with positive prognostic effects to improve the quality of life in patients affected by heart disorders.

#### 5.5.1 Preclinical studies

Early experimental studies demonstrated the antihypertensive effect mediated by renal denervation in animal models with nephrectomy or metabolic syndrome (Kassab et al., 1995; Norman et al., 1984-; O'Hagan et al., 1990). Renal denervation in a cardiac post-ischemic model significantly improved the postprandial sodium excretion along with cardiac function and baroreflex responses, favoring a decrease in AT1 receptor density, thus counteracting the cardiovascular adverse remodeling (Villarreal et al., 1994; Airaksinen et al., 1999; Clayton et al., 2011; Lataro et al., 2017). Interestingly, the positive effect of renal denervation has recently been reported in cardiac neuronal settings (Hu et al., 2014b). Notably, this study proposed separating the renal artery from the abdominal aorta in a specifically analyzed population of rats. The authors have described the abolition of RAAS hyperactivity, with the preservation of renal function, without any implications on the cardiovascular system. Interestingly, ventricular remodeling improvement was even more relevant than with typical drugs against HF (beta antagonists, ACE inhibitors, and sartans). Indeed, it seems that a “renal nervous silencing” would exercise an indirect effect on the heart rate through renal adrenergic fibers annulment, which in turn inhibits sympathetic control. In addition, an increase in ventricular dynamic responses, connected to the reduction of both B-type natriuretic peptide (BNP) and norepinephrine levels, have been obtained, with the consequential restoration of cardiac autonomic control. According to the authors, this technique might represent an alternative therapy for HF patients who are not adequately controlled with classical anti-CHF drugs.

#### 5.5.2 Clinical trials

Initial clinical investigations demonstrated antihypertensive effects of splanchniectomy (surgical paralumbar sympathectomy technique), associated with a reduction in mortality risk in severely hypertensive patients (Page, 1934; Page and Heuer, 1935; Smithwick and Thompson, 1953). After numerous attempts, the European Commission approved the minimally invasive procedures for renal denervation in clinical practice for the treatment of cardiac disorders (Böhm et al., 2013). Renal denervation is achieved through the administration of high-frequency energy, thus inducing the annulment of adrenergic fibers in the adventitia of the renal arteries (Krum et al., 2011b). Interestingly, a proof-of-principle multicenter study showed a significant reduction in blood pressure assessed in subjects with uncontrolled hypertension through catheter-based renal denervation (Krum et al., 2009). A decline in renal catecholamine spillover was observed, along with a parallel lowering in systemic catecholamine amounts, thus contributing to the reduction of central SNS activity. (Krum et al., 2009; Schlaich et al., 2009). Of note, renal denervation in patients affected by refractory hypertension induced an improvement in diastolic function recovery accompanied by both LV mass and blood pressure reduction (Brandt et al., 2012; McMurray et al., 2012). The majority of clinical trials for renal denervation in HF were accomplished in patients with symptomatic heart failure and reduced LV ejection fraction (HFrEF; LVEF ≤40%). In the Renal Artery Denervation in Chronic Heart Failure (REACH) study (Davies et al., 2013), preliminary 6-month follow-up results reported no significant decrease in blood pressure and no syncope or hypotension in seven HF patients with HFrEF. However, improvement in the 6-min walking distance analysis and quality-of-life parameters was detected. Larger randomized trials, such as the SYMPLICITY-HF study (Renal Denervation in Patients with Chronic Heart Failure and Renal Impairment, NCT01392196) and the RSD4CHF trial (Renal Sympathetic Denervation for Patients with Chronic Heart Failure, NCT01790906) have confirmed the REACH findings in a wider systolic HF cohort (SymplicityHF, available at: https://clinicaltrials.gov/ct2/show/NCT01392196. Accessed 1 March 2015 (RSD4CHF), Available at: https://www.clinicaltrials.gov/ct2/show/NCT017 90906. Accessed 1 March 2015). These randomized trials demonstrated the safety and efficacy of renal sympathetic denervation as a well-tolerated treatment to delay the progression of chronic HF, effectively reducing all-cause mortality. Renal denervation significantly decreased LV hypertrophic maladaptive remodeling in patients with resistant hypertension, thus allowing the use of renal denervation also in the HF population with preserved LV ejection fraction (HFpEF; LVEF ≥50%) (Brandt et al., 2012; Zile and Little, 2012). The antihypertrophic effects mediated by renal denervation in refractory hypertensive patients were partially due to a modulation of blood pressure with improvement in myocardial load, but also related to modifications in the control of the autonomic system (Zile and Little, 2012). Of interest, the DIASTOLE trial (Denervation of the Renal Sympathetic Nerves in Heart Failure with Normal LV Ejection Fraction, NCT01583881) is a multicenter, randomized controlled study that evaluated the effects of renal denervation in HF patients with normal LVEF (HFnEF) and hypertension. The effects of renal denervation on the myocardial remodeling parameters (LVEF modifications and LV mass) were analyzed, together with the assessment of MIBG (uptake and washout), BNP levels, blood pressure, and quality of life scores (Verloop et al., 2013). Similarly, in the ongoing SWAN-HF study (Renal Sympathetic Modification in Patients with Heart Failure, NCT01402726), the primary objective was to investigate the incidence of composite cardiovascular episodes after renal ablation with sympathetic modification through THERMOCOOL® catheter in the HF cohort (Available at: https://www.clinicaltrials.gov/ct2/show/study/NCT01402726. Accessed 12 April 2015). Renal denervation showed important beneficial effects in the modulation of numerous comorbidities involved in the stimulation of the sympathetic system and liable for the exacerbation of HF pathological mechanisms, such as glucose tolerance, insulin levels, and HOMA indices in hypertensive patients affected by metabolic disorders (Mahfoud et al., 2011; Witkowski et al., 2011), associated with a significant amelioration of renal hemodynamic and microalbuminuria in patients with resistant hypertension (Mahfoud et al., 2012). In addition, renal denervation promoted antiarrhythmic effects along with a significant improvement in vascular stiffness in the db/db mouse model with HFpEF (Reil et al., 2013). Albeit the safety and effectiveness of renal denervation will have to be tested properly in lagers trials, this procedure showed beneficial cardiac effects and was well tolerated in HF patients. However, the co-presence of risk factors and cardiovascular comorbidities, together with heterogeneous responses of the adrenergic renal system will need to be evaluated before applying renal denervation as a therapy in the HF population.

### 5.6 Vagal nerve stimulation

#### 5.6.1 Preclinical studies

Experimental evidence highlighted the efficacy and beneficial effects mediated by VNS as a novel approach for the treatment of several cardiovascular diseases (Buckley et al., 2015; Schwartz et al., 2015). Specifically, vagus neurostimulation in an endotoxemia setting induced an anti-inflammatory response, inhibiting T cell activation, and mediating a hemodynamic effect, thus reducing the hypertensive risk (Carnevale et al., 2016; Carnevale et al., 2020). Along this line, recent evidence has demonstrated the modulation of the peripheral immunological responses mediated by insular cortex neurons through vagal control stations, the DMV, and rostral ventrolateral medulla (RVLM), involved in ANS regulation (Koren et al., 2021). Moreover, vagal nerve stimulation mitigated acute kidney injury (AKI) and reduced TNF-α systemic levels in a mouse model, through α7 nicotinic acetylcholine receptors (α7nAChRs) positive splenocytes (Inoue et al., 2016). In a cardiac post-ischemic rat model, Li and colleagues described a significant improvement in cardiac remodeling and ventricular function after 6 weeks of right VNS, associated with decreased NE and BNP circulating levels and increase in survival rates (Li et al., 2004). Similarly, dogs with ventricular tachypacing-mediated HF, treated with right VNS showed enhanced LV performance and mitigated HF-induced cardiovascular remodeling (Zhang et al., 2009). Additionally, Beaumont demonstrated that chronic VNS induced an improvement in cardiac maladaptive hypertrophic remodeling and neuronal impairment in a guinea pig model of aortic constriction-induced chronic pressure-overload (TAC model) (Beaumont et al., 2016). Overall, these findings hint at the fact that vagal neuromodulation affects may be related to ANS homeostasis restoration, anti-inflammatory effects, alteration of metabolic processes, and regulation of apoptotic signaling pathways (Zhang et al., 2009; Zhao et al., 2013; Beaumont et al., 2016). The VNS approach showed anti-arrhythmic beneficial effects also in cardiac MI models. Notably, in a cardiac post-MI feline model, bilateral vagotomy or atropine administration reduced ventricular fibrillation (VF) episodes and the mortality rate (Corr and Gillis, 1974). Similarly, vagal hypertone diminished the risk of VF events in a canine model that underwent myocardial infarction (Billman et al., 1982). In addition, in dogs predisposed to VF and placed on a daily exercise program, Billman and colleagues have shown a significant enhancement in baroreflexes responses related to ANS tone improvement, resulting in a sensible reduction in ventricular arrhythmias (Billman et al., 1984). Along this line, VNS counteracted ventricular tachyarrhythmic attacks in a cardiac post-ischemic murine model, and this effect was abolished by atropine pretreatment (Ando et al., 2005). Of note, a recent porcine MI study suggested protective effects of vagal neuromodulation, which reduced the risk of VF onset in the scar-border zone, recognized clinically as an arrhythmogenic substrate (Rajendran et al., 2016). Overall, these data evidenced the potential benefits of VNS for the improvement of vagal reflexes, stabilization of the heart rhythm and control of the “storming of electric impulses” that occurs during myocardial infarction. At the cardiac level, vagal postganglionic fibers activate M2 cardiomyocyte receptors, through acetylcholine release, thus determining negative chronotropic, dromotropic, and, in part, inotropic effects (Jänig, 2016). Since vagal afferents modulate vasorelaxation through the activation of nitric oxide signaling pathways, VNS modulates coronary blood flow, improving heart rate and aortic pressure, partially attenuated by atropine pretreatment (Feigl, 1969). Lastly, vagal neuromodulation improves cardiac ANS tone attenuating heart failure progression through the reduction of pro-inflammatory cytokine expression and both NE and AT- II circulating levels (Tracey, 2007; Zhang et al., 2009). Moreover, two main neurotrophic factors (i.e., NGF and BDNF) resulted significantly influenced by VNS. All neurotrophins have been located in the nodose ganglion and in nervous central stations, stimulated by vagal inputs (Helke et al., 1998). Interestingly, vagotomy affects the distribution of NGF and BDNF and their receptors in the brain vagal efferent sites (O'Leary et al., 2018), thus highlighting the crucial role played by neurotrophic factors in the homeostasis of vagal transmission. Additionally, based on protocols and parameters of vagal stimulation, modifications in NGF and BDNF expression in the brain parenchyma have been reported (Follesa et al., 2007). Thus, NTFs production and secretion may mirror the stimulation degree of the vagus nerve, confirming the potential benefits of establishing adequate protocols for VNS application. VNS may also modulate neurotrophins expression locally along with their receptors, thus stimulating immune responses with anti-inflammatory effects (Ay et al., 2016; Cheyuo et al., 2011; Pöyhönen et al., 2019). Lastly, it is plausible that vagal neurostimulation may affect the NTFs maturation processes, thus modulating the expression of the neurotrophins that contribute to trophic effects in the brain (Davies, 1997). Thus, neurotrophins may modulate brain neurogenesis and neuronal plasticity through vagal neurostimulation (Biggio et al., 2009). Accordingly, the circulating levels of NGF and BDNF and their daily changes might act as potential biomarkers for VNS application. In sum, vagal nerve stimulation can affect cerebral NTFs activity through control of NGF and BDNF production and secretion in an activity-dependent manner, neurotrophic modulation of the neurohormonal axis, and neurotrophic-dependent neurogenesis and plasticity. Based on the interconnection between vagal innervation and neurotrophic signaling pathway, we speculate that VNS may represent a novel potential therapeutic strategy to mitigate NTF impairment and neuronal dysfunction in both neurodegenerative and cardiovascular disorders.

#### 5.6.2 Clinical studies

Originally, VNS was used as a treatment for depression and refractory epilepsy episodes (Aaronson et al., 2013; Arle et al., 2016). Although the anti-convulsant effects of vagal neurostimulation are still undefined, numerous clinical trials have documented the reduction in both epileptic attacks and anti-convulsant agents’ usage (Ben-Menachem et al., 2015). The Food and Drug Administration recently approved implantable pulse generators for seizure treatment, also encouraging the application of this technique for the treatment of other diseases (Ben-Menachem et al., 2015). Based on these premises, some clinical studies started to investigate the effectiveness and potential benefits of VNS as a novel therapy for cardiac disorders (Schwartz et al., 2008; De Ferrari et al., 2011; Premchand et al., 2014; Gold et al., 2016; De Ferrari et al., 2017). Initially, VNS was administered along with canonical pharmacological therapies, showing divergent effects in HF patients (Anand et al., 2020). Other randomized controlled trials have explored the safety and cardioprotective effect of vagal nerve stimulation. Notably, in an open-label randomized trial, Autonomic Neural Therapy to Enhance Myocardial Function in Heart Failure (ANTHEM-HF), VNS was assessed in 60 patients with NYHA functional class II- III symptoms and reduced ejection fraction (LVEF ≤40%) (Premchand et al., 2016; DiCarlo et al., 2018; Konstam et al., 2019; Sharma et al., 2021). Among recent trials, only the ANTHEM-HF study evidenced the beneficial effects of VNS for HF (Anand et al., 2020). Interestingly, two other randomized controlled trials have investigated the modulation of VNS-mediated cardiovascular function in HF subjects, including NEural Cardiac TherApy for HF (NECTAR-HF) and INcrease Of VAgal TonE in Heart Failure (INOVATE-HF) (Hauptman et al., 2012; Premchand et al., 2014; Zannad et al., 2015; Gold et al., 2016). The NECTAR-HF trial analyzed 96 HF patients with left ventricle chamber dilation and HFrEF (LV ejection fraction ≤35%) (Zannad et al., 2015). Initial data collected after 6 months of the study did not show any significant difference in the primary specified outcome of LV end-systolic internal diameters. Additionally, VNS treatment did not improve quality-of-life endpoints nor induced any relevant changes in NYHA functional class (Zannad et al., 2015). In the NECTAR-HF trial, however, the HF cohort was heterogeneous, and lack of specificity in electric stimuli and poor modulation of vagal cardiac efferent fibers were recognized limitations (De Ferrari et al., 2017; Anand et al., 2020). The INOVATE-HF multinational, randomized trial enrolled 707 patients with chronic HF, NYHA functional class III symptoms, LV dilation, and reduced LV ejection fraction (LVEF ≤40%). VNS was achieved using an R-wave-triggered pulse, resulting in a significant blockage of the vagal afferent inputs, but accompanied by stimulation of its efferent terminals (Gold et al., 2016). The results of the INOVATE-HF trial have evidenced no significant reduction in mortality rate or HF episodes after VNS. However, vagal nerve modulation positively impacted on NYHA functional class, 6-min walking distance test, and quality-of-life outcomes (*p* < 0.05), although no difference was appreciated in LV end-systolic volume measurements (*p* = 0.49) (Gold et al., 2016).

The contrasting results that emerged in these clinical trials may be related to heterogeneity in the HF population and VNS protocol applied (Anand et al., 2020). However, since early trials have had mixed results, the role of VNS in heart failure remains to be proven. Indeed, more studies will be needed to confirm the efficacy of this approach.

The development of specific biomarkers to monitor the effects of VNS on cholinergic tone, adrenergic hyperactivity, and control the impaired afferent system, will allow to determine whether vagal neurostimulation has clinical advantages over approved therapies.

## 6 Conclusion

Based on this rich body of evidence, we postulate that innovative therapeutic approaches are needed to promote the reparative mechanisms in the heart after injury, facilitate the restoration of the autonomic nervous system, and preserve neurotrophic signaling pathway homeostasis in the brain/heart axis. In this regard, more research is needed to dissect the physiological role played by ANS stimulation and neurotrophins in the heart tissue. This new knowledge will positively impact both neurodegenerative disorders as well as different cardiovascular diseases. Indeed, neuromodulation approaches could represent a key solution to counteract the cardiac nervous system remodeling, which leads to the severe myocardial denervation and lethal cardiac disorders (such as AF, MI, HF). Although the application of neurotrophins in cardiovascular diseases is still debated and technical concerns have been raised about their administration, parallel findings demonstrate the key role played by NTFs in the modulation of the brain/heart axis, with possible preventive value. Since cardiovascular disease is known to increase AD/dementia risk and pathology (Carey and Fossati, 2022), the proposed strategies may also reveal to have invaluable therapeutic or preventive potential in AD, as well as vascular dementias and related neurodegenerative disorders.

Achieving a better understanding of the poorly described role of the cardiovascular neuro-signaling pathway and the ANS in both heart disorders and dementias, while expanding our knowledge of the disease mechanisms, will allow the development of novel biomarkers and therapeutic strategies to correct or delay the adverse cardiovascular remodeling, improving the prognosis, pathology and quality of life in patients with heart disease, AD, or different types of dementias.
